# Clinical and radiological outcomes of the multilevel Ponte osteotomy with posterior selective segmental pedicle screw constructs to treat adolescent thoracic idiopathic scoliosis

**DOI:** 10.1186/s13018-018-1001-0

**Published:** 2018-11-29

**Authors:** Jing Feng, Juan Zhou, Mi Huang, Ping Xia, Wei Liu

**Affiliations:** Department of Orthopaedics, First Hospital of Wuhan, No. 215 Zhongshan Road, Wuhan, 430022 China

**Keywords:** Ponte osteotomy, Selective segmental pedicle screw, Adolescent thoracic idiopathic scoliosis

## Abstract

**Background:**

To compare the clinical and radiological outcomes of the surgical correction of Lenke type 1 to 4 scoliosis by using a multilevel Ponte osteotomy procedure with posterior selective segmental pedicle screw constructs or posterior release and selective segmental pedicle screw constructs only in patients with adolescent thoracic idiopathic scoliosis.

**Methods:**

Retrospective analysis of 65 patients, 32 treated with the multilevel Ponte procedure (Group A) and 33 with posterior soft tissue release only (Group B). The groups were compared with regard to the change in spinal alignment from preoperative to postoperative assessment and over the follow-up period.

**Results:**

A correction rate of the main thoracic curve of 63.9 ± 4.5% was obtained for group A and 65.2 ± 2.4% for group B (*P* = 0.17). However, the Cincinnati correction index was greater for group A (1.8 ± 0.3) than that for group B (1.4 ± 0.2, *P* < 0.001), with a smaller change in angle over the period from 1 week postoperatively to the 2-year follow-up (*P* < 0.05). The operative time, volume of blood loss, and volume of transfusion were greater for group A than for group B (*P* < 0.05).

**Conclusion:**

The multilevel Ponte osteotomy procedure, with posterior selective segmental pedicle screw constructs, improves the Cincinnati correction index and restores the thoracic kyphosis in patients with adolescent thoracic idiopathic scoliosis.

## Background

Idiopathic scoliosis is one of the most common types of spinal scoliotic deformity. Of unknown etiology, idiopathic scoliosis usually develops in adolescents and, as such, is generally referred to as adolescent idiopathic scoliosis (AIS). The main goal of the surgical treatment of AIS is to correct the spinal deformity, restore the coronal and sagittal balance of the spine, prevent malformations and progression, and improve appearance and cardiopulmonary function. In recent years, the Ponte osteotomy procedure has been increasingly used as a corrective approach for AIS, although there is controversy around the clinical effectiveness of this procedure. Samdani et al. [1] evaluated the outcomes of the surgical correction of Lenke type 1A and 1B curvatures using pedicle screws, with or without a Ponte osteotomy, among 191 patients with AIS. Based on their findings, these authors concluded that the Ponte osteotomy procedure improves the correction of the structural deformity of the stiff and lordotic curves along all three anatomical planes. In a similar manner, Shah et al. [2] proposed that a Ponte osteotomy could significantly improve the radiographic parameters of the coronal and sagittal balance of AIS. In contrast, in a small cohort study, Halanski et al. [3] reported that the Ponte osteotomy did not significantly improve the coronal and sagittal alignment of the thoracic curve, while prolonging operative time and increasing the volume of bleeding. To address existing controversy regarding the role of Ponte osteotomy in the correction of AIS, we undertook a retrospective analysis to compare the clinical and radiographic outcomes of surgical correction of Lenke type 1–4 curves using multilevel Ponte osteotomies combined with posterior selective segmental pedicle screw constructs or posterior release and selective segmental pedicle screw constructs only.

## Materials and methods

### Patient selection

The patients were retrospectively identified from our surgical database using the following inclusion criteria: Lenke type 1 to 4 scoliosis, based on preoperative imaging (radiographs, computed tomography, and magnetic resonance); corrective surgery using either the multilevel Ponte osteotomy procedure combined with posterior selective segmental pedicle screw constructs or posterior selective segmental pedicle screw constructs only; and availability of complete clinical data throughout the follow-up period. Sixty-five patients met the inclusion criteria and were enrolled in the study. Among these, 32 had been treated using multilevel Ponte osteotomy (group A) and 33 using soft tissue release only (group B).

Group A included 10 males and 22 females, 13 to 18 years of age (mean, 15.1 ± 1.9 years), with a disease course of 2–6 years (mean, 3.5 ± 1.1 years) and a Risser sign of 3.3 ± 0.9. After corrective surgery, thoracic kyphosis was decreased in 11 cases, within normal limits in 18 cases and increased in 3 cases. All patients were followed-up, on average, for 24.2 months (range, 18–30 months).

Group B included 10 males and 23 females, 13 to 19 years of age (mean, 15.7 ± 1.9 years), with a disease course of 2–6 years (mean, 3.3 ± 1.3 years) and a Risser sign of 3.1 ± 1.0. Thoracic kyphosis was decreased in 11 cases, normal in 19 cases, and increased in 4 cases. All patients were followed-up, on average, for 23.9 months (range, 18–30 months).

Prior to surgery, full-length spinal radiographs (standing anterior-posterior and lateral views), computed tomography (CT) and magnetic resonance (MR) images of the spinal region of interest for correction, echocardiography, B-mode ultrasonography of the urinary system, and pulmonary function testing were routinely obtained. No evidence of pathology or structural abnormalities of the spinal canal and neural structures were identified in any patient, and all patients had unremarkable assessments of the cardiorespiratory, urinary, and renal systems.

The distribution of sex, age, disease course, Risser sign, number of segments fused, postoperative coronal Cobb angle, and thoracic kyphosis were comparable between the two groups (*P* > 0.05; Table 1).

**Table 1:** Basic information of patients in two groups

		Patients (F)	Age	Course of disease	Risser	Percentage flexibility (%)	Pinning density	Fused segments
*A*	32	22	16.1 ± 1.9	3.5 ± 1.1	3.3 ± 0.9	34.7 ± 7.9	66.1 ± 7.7	10.3 ± 2.0
*B*	33	23	15.7 ± 1.9	3.3 ± 1.3	3.1 ± 1.0	47.5 ± 6.8	66.4 ± 7.3	9.6 ± 2.3
		*×*^2^ = 0.007	*t* = 0.703	*t* = 0.766	*t* = 0.830	*t* = −6.998	*t* = −0.128	*t* = 1.416
		*P* = 0.934	*P* = 0.484	*P* = 0.447	*P* = 0.410	*P* = 0.000	*P* = 0.898	*P* = 0.162

### Surgical procedure

For group A, after administration of general anesthesia and intubation, the patients were placed in the prone position with the abdomen suspended. A midline posterior incision was performed to expose the posterior structures of the spine. Subperiostial tissues were dissected to expose the laminae, articular processes, and transverse processes. A pedicle screw was manually inserted into the appropriate vertebrae based on the flexibility of the scoliosis and the number of interval vertebrae: upper vertebral body of the top vertebra of the scoliosis and the adjacent stable vertebra or between the two top vertebrae of the scoliosis and the adjacent stable vertebra. A C-arm was used to visualize the length of penetration and location of the pedicle screw.

We subsequently proceeded with the release of the posterior structures of the spine using a rongeur to remove the spinous processes, interspinous ligaments, and supraspinous ligaments along the root. The yellow ligament was also completely removed using a gun-like rongeur up to the anterior surface of the facet joints. Subsequently, the facet joints and inferior articular processes were completely resected bilaterally until the intervertebral opening was completely open to retain the integrity of the pedicle. We also resected the edge of the upper and lower lamina to achieve a uniform width of the lamina window. A Ponte osteotomy was then performed at each segment of the thoracic curvature. The spinal rod, which had been prebent according to the degree of curvature of the scoliosis and the sagittal physiological curve, was placed on the concave side of the thoracic curve, with the length and orientation adjusted to achieve appropriate distraction up to the top vertebra. A second spinal rod was then inserted on the convex side and tightened. After adequate correction of the spinal alignment was confirmed, the surgical field was rinsed using a flushing gun. The cortical bone of the posterior laminae was then grounded and treated with a mixture of autogenic and allogeneic bone. A drainage tube was placed in situ*,* and the incision was closed. All surgeries were performed under monitoring of the spinal cord function, and only autologous blood transfusion was used, as needed.

For group B, the same surgical procedure as for group A was performed, with the exception that the interspinal and intertransverse ligaments and joint capsules were completely resected by cauterization and using a rongeur, but without performing a posterior column osteotomy.

### Outcome measures

The following intraoperative variables were recorded for analysis: operative time, volume of blood loss, units of blood transfused, and surgery-related complications. Full-length spinal radiographs (standing anterior-posterior, lateral, and bending views) were obtained preoperatively to evaluate the degree of deformity and stiffness. The radiographs were repeated at the time of postoperative discharge and at each follow-up for comparison to preoperative values. The Cobb method was used to measure the coronal Cobb angle of the thoracic curve, with the sagittal Cobb angle used to measure the thoracic kyphosis, from the superior endplate of T5 to the inferior endplate of T12. The rate of correction was calculated as follows: preoperative Cobb angle—postoperative Cobb angle)/preoperative Cobb angle × 100%. The Cincinnati correction index (CCI) was calculated as follows: postoperative correction degree—preoperative Cobb angle in standing position/preoperative convex Cobb angle in the supine position with lateral bending—preoperative Cobb angle [4].

### Statistical methods

All statistical analyses were performed using SPSS (version 19.0). The means ± standard deviation was calculated for all variables, with between-group differences evaluated using an independent sample *t* test or chi-squared test as appropriate for the date type. A *P* value < 0.05 was considered statistically significant.

## Results

### Surgical outcomes

All procedures were performed by the same surgeon. The operative variables are reported in Table 2 and summarized as follows. All operative variables were greater for group A than for group B (*P* < 0.05): operative time, 243 ± 12 min versus 196 ± 10 min; intraoperative volume of blood loss, 952 ± 124 ml versus 772 ± 65 ml; and intraoperative blood transfusion, 837 ± 289 ml versus 610 ± 160 ml. With regard to surgery-related complications, there was one incidence of hemopneumothorax in group A, with two cases of superficial tissue infection in group B. These complications improved with symptomatic and supportive treatments. There was no incidence of neurological injury or failure of the internal fixation in our case series.

**Table 2:** Intraoperative and postoperative complications of the two groups

Group	Operation time	The amount of bleeding	Blood transfusion	Complication
*A*	(243 ± 12) min	(952 ± 124) ml	(837 ± 289) ml	1 (3.1%)
*B*	(196 ± 10) min	(772 ± 65) ml	(610 ± 160) ml	2 (6.1%)
	*t* = 17.525	*t* = 7.314	*t* = 3.895	*×*^2^ = 0.289
	*P* = 0.000	*P* = 0.000	*P* = 0.000	*P* = 0.591

### Spinal correction: coronal plane

The coronal plane Cobb angles are reported in Table 3. The preoperative and postoperative coronal Cobb angles were comparable for both groups, with equivalent curve correction achieved, 63.9 ± 4.5% for group A and 65.2 ± 2.4% for group B (*t* = − 1.383, *P* = 0.170). The postoperative CCI was greater for group A (1.8 ± 0.3) than for group B (1.4 ± 0.2); this between-group difference was significant (*t* = 6.504, *P* < 0.001). The coronal plane Cobb angle was comparable between the two groups at the last follow-up, 21.1° ± 1.5° for group A and 21.4° ± 2.9° for group B (*t* = − 0.526, *P* = 0.062).

**Table 3:** Comparison of the Cobb angles of coronal major thoracic curve of patience in two groups before, after the surgery, and at the last follow-up

Cobb angel	Standing position (pre)	Bending (pre)	Standing position (post)	Correction rate	CCI	Standing position
*A*	57.6° ± 10.3°	36.8° ± 7.4°	19.9° ± 1.6°	(63.9 ± 4.5)%	1.8 ± 0.3	21.1° ± 1.5°
*B*	56.1° ± 8.9°	29.4° ± 6.3°	19.6° ± 2.9°	(65.2 ± 2.4)%	1.4 ± 0.2	21.4° ± 2.9°
*t*	0.632	4.382	0.359	− 1.383	6.504	− 0.526
*P*	0.530	0.000	0.720	0.170	0.000	0.062

### Spinal correction: sagittal plane

**Fig. 1:**
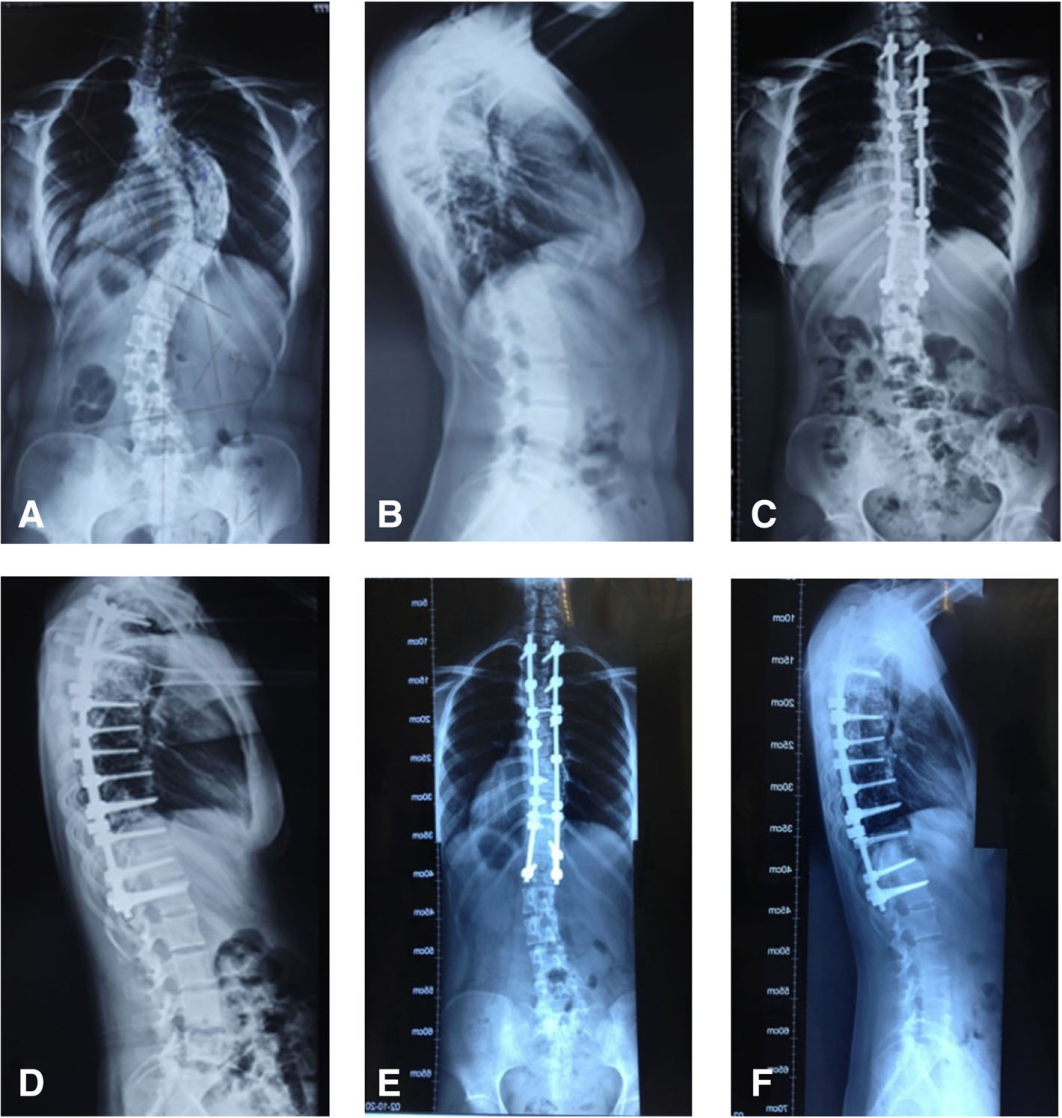
Typical case from group A. Spinal radiographs for an 18-year-old female patient with Lenke type 4 idiopathic scoliosis, which was corrected using a multilevel Ponte osteotomy procedure combined with posterior segmental selective pedicle screw implantation. **a** Preoperative posterior-anterior radiograph showing a coronal Cobb angle of the major thoracic curve of 71°; **b** preoperative lateral radiograph showing a thoracic kyphosis angle of 9°; **c** anterior-posterior radiograph at 1 week postoperatively, showing a coronal plane Cobb angle of the major thoracic curve of 19° (correction rate, 73.2%; CCI, 4.73); **d** lateral radiograph at 1 week postoperatively, showing a thoracic kyphosis angle of 17°; **e** anterior-posterior radiograph at 2 years postoperatively, showing a coronal plane Cobb angle of the main thoracic curve of 20°; and **f** lateral radiograph at 2 years postoperatively, showing a thoracic kyphosis angle of 18°

**Fig. 2:**
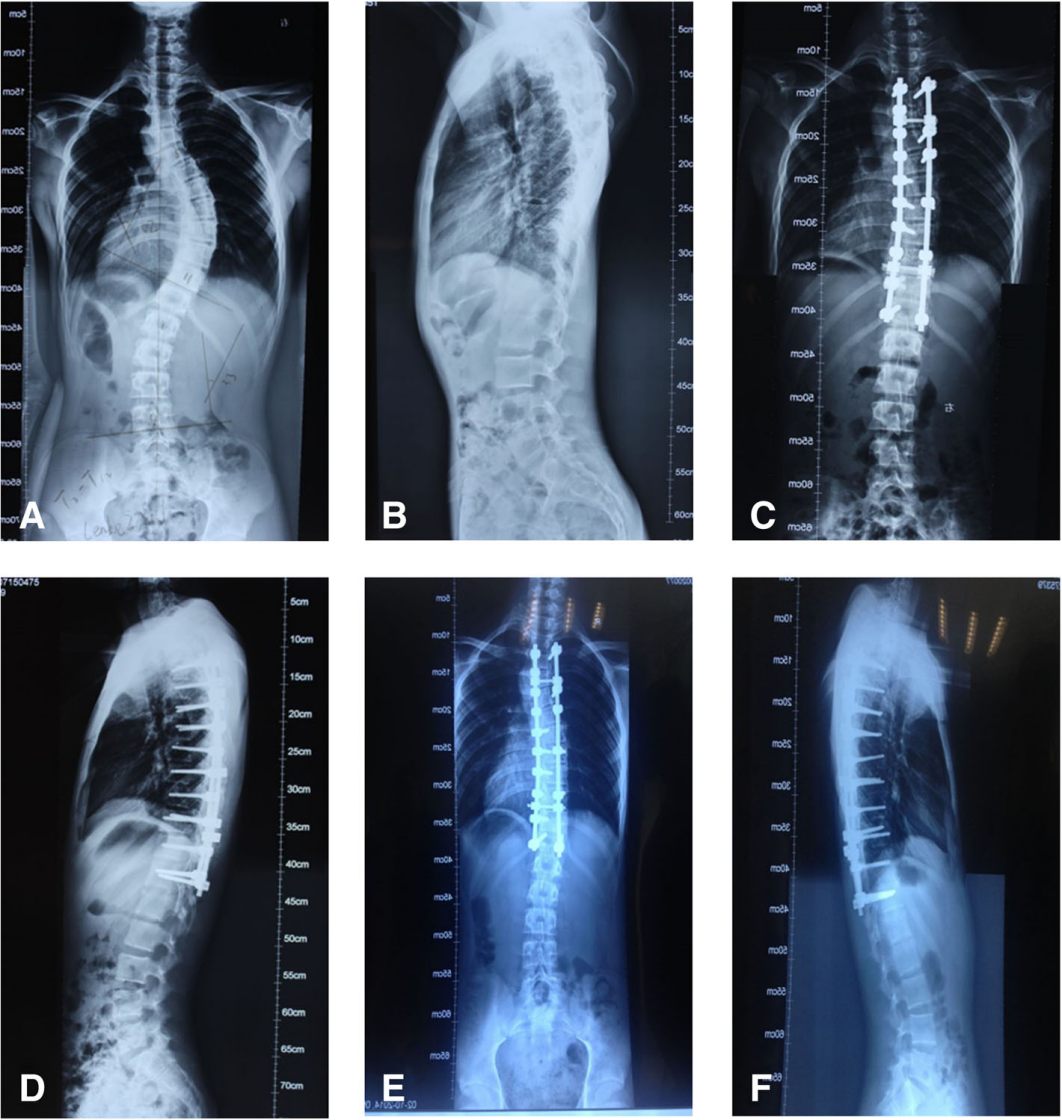
Typical case from group B. Spinal radiographs for a 14-year-old male patient with a preoperative diagnosis of Lenke type 2 idiopathic scoliosis, which was corrected by soft tissue release only, combined with selective posterior segmental pedicle screws. **a** Preoperative anterior-posterior radiograph, showing a coronal Cobb angle of the main thoracic curve of 52°; **b** preoperative lateral radiograph, showing a thoracic kyphosis angle of 4°; **c** anterior-posterior radiograph obtained 1 week postoperatively, showing a coronal plane Cobb angle of the major thoracic curve of 15° (correction rate, 71.2%; CCI, 1.12); **d** lateral radiograph obtained 1 week postoperatively, showing a thoracic kyphosis angle of 8°; **e** anterior-posterior radiograph at 2 years postoperatively, showing a coronal plane Cobb angle of the main thoracic curve of 17°; and **f** lateral radiograph at 2 years postoperatively, showing a thoracic kyphosis angle of 9°

The Cobb angles for the thoracic kyphosis (T5–T12) are reported in Table 4. The patients in both groups were classified based on the postoperative thoracic kyphosis angle as follows: increased thoracic kyphosis (> 40°), normal thoracic kyphosis (10°~40°), and decreased thoracic kyphosis (< 10°). A greater rate of correction of the thoracic kyphosis was achieved in group A than that in group B (*P* < 0.05). A typical case for both procedures is shown in Figs. 1 and 2.

**Table 4:** Comparison of the sagittal thoracic kyphotic of patients in different groups before, after the surgery and at the last follow-up

Group/thoracic lordosis angle	*n*	Preoperative	Postoperative	Last follow-up	Correction angle
Hyper group					
*A*	3	45.3° ± 5.5°	27.0° ± 2.0°	25.7° ± 1.5°	18.3° ± 2.5°
*B*	4	43.0° ± 1.4°	30.5° ± 1.3°	30.3 ± 1.0°	12.5° ± 1.3°
*t*		1.740	− 2.842	− 4.927	4.063 s
*P*		0.098	0.036	0.004	0.010
Normal group					
*A*	18	21.3° ± 6.7°	28.4° ± 4.6°	27.3° ± 4.4°	− 7.1° ± 10.3°
*B*	19	23.7° ± 5.5°	24.4° ± 6.2°	23.6° ± 6.3°	− 0.7° ± 4.6°
*t*		− 1.163	2.246	2.089	− 2.413
*P*		0.253	0.031	0.044	0.024
Hypo group					
*A*	11	7.3° ± 1.9°	18.4° ± 3.2°	17.5° ± 3.2°	− 11.1° ± 2.9°
*B*	11	8.0° ± 1.4°	11.5° ± 2.4°	10.9° ± 2.5°	− 3.5° ± 2.2°
*t*		− 1.000	5.706	5.302	− 6.877
*P*		0.329	0.000	0.000	0.000

## Discussion

### Application of selective segmental pedicle screw constructs for the treatment of AIS

Increasingly, pedicle screw fixation is used for correcting spinal alignment in young patients with AIS. Pedicle screws can reduce stress concentration and improve the coronal and sagittal balance of the spinal alignment, which lowers the risk of distraction, compression, movement, and rotation of the spinal rod implants and reduces the postoperative crankshaft phenomenon [5, 6]. However, the placement of pedicle screws increases the risk of spinal cord injury, especially at the superior spinal levels of the thoracic spine. Moreover, the cost of pedicle screws will inevitably increase patient medical expenses, which is opposed to the existing level of medical care coverage and national initiatives of China. Therefore, for patients with flexible AIS, selective segmental pedicle screw fixation can achieve comparable results at a lower cost. In our study group, for all 65 patients, the flexibility of the scoliotic curve was > 30%, and therefore, segmental pedicle screw fixation was used, without incidence of pedicle rupture, pulled or broken nails or rods, or spinal cord injury. We achieved a correction rate of > 63%, which is similar to the rate previously reported for full-segment pedicle screw fixation [7, 8].

### Effect of multilevel Ponte osteotomy for correction of the coronal plane alignment

The Ponte osteotomy technique includes the release of the intervertebral bony structure of the posterior column. The technique was first described by Alberto Ponte in 1984 and used for the surgical treatment of Scheuermann’s disease, with complete resection of the spinous ligaments, interspinous ligaments, and the yellow ligament, as well as the complete resection of the spinous processes, the lower articular process of the upper vertebral body, and the upper articular process of the lower vertebral body to completely open the intervertebral foramen [9]. The multilevel Ponte osteotomy procedure is used between continuous multiple segments of the posterior column, with the posterior release of the scoliosis improving the flexibility of the spine, which is more conducive to the correction of coronal plane alignment. However, the efficacy of surgical effectiveness remains controversial. Halanski et al. [3] argue that the multilevel Ponte osteotomy procedure does not improve the correction rate of coronal spinal alignment in idiopathic scoliosis. In contrast, Shah et al. [2] demonstrated that the Ponte osteotomy procedure significantly improves both coronal and sagittal plane alignment in patients with juvenile idiopathic thoracic scoliosis. These studies, however, did not consider the effects of differences in preoperative spinal flexibility on postoperative outcomes.

### AIS 窗体顶端

AIS is a complex spinal deformity for which postoperative correction outcomes can be influenced by multiple factors, including the degree of preoperative flexibility of the spine, the density of the bone in which the nails are implanted, the degree of intraoperative soft tissue release, and the position of stable vertebra. These modifying factors need to be controlled when comparing the outcomes of different procedures. To control the preoperative flexibility of the spine prior to correction, we used the CCI [4] to specifically evaluate the effect of the multilevel Ponte osteotomy procedure. We demonstrate the sensitivity of the CCI in our study, compared to using only the Cobb angle. Specifically, while the correction rate based on the Cobb angle was equivalent for groups A and B (*P* = 0.170), the CCI was significantly greater for group A than for B (*P* < 0.001), demonstrating the orthopedic superiority of the Ponte osteotomy procedure compared to the posterior release of soft tissues only. For patients with poor flexibility in group A, the Ponte osteotomy was performed at each segment of the structural main thoracic curve, and a wider posterior release was performed. This procedure improved the intraoperative flexibility of the spine, allowing us to obtain a correction rate of the Cobb angle that was comparable to that of group B, and improved the CCI.

### Effect of multilevel Ponte osteotomy for the correction of the sagittal plane alignment

Many patients with AIS have inadequate native thoracic kyphosis or even show lordosis of the thoracic spine. Therefore, one of the aims of AIS treatment is to obtain physiological kyphosis. The correction of physiological kyphosis is influenced by many factors during orthopedic surgery. Cidambi [9] et al. considered that prebending of the spinal rod applied to the concave side favors a greater correction of thoracic kyphosis and reduces the degree of loss of the correction after surgery. Fletcher et al. [10] also considered that the diameter of the rod can significantly impact the correction of the sagittal alignment of the thoracic spine; a rod with a diameter of < 5.5 mm is associated with a higher risk for the loss of correction of kyphosis postoperatively, as well as being inadequate for the correction of native kyphosis.

The choice of the internal fixation system also has a significant impact on the correction of thoracic kyphosis. Watanabe et al. [11] reported that after correction of the coronal deformity of the AIS through adjustment of the rotation of the spinal segment via a pedicle screw system, it might be difficult to correct thoracic kyphosis. However, the specific effectiveness of the Ponte osteotomy in correcting the sagittal alignment of the spine in patients with AIS is still controversial. In our case series, for patients who had increased thoracic kyphosis, the Ponte osteotomy was effective in reducing the degree of thoracic kyphosis. Similarly, for patients who had a flat back deformity (thoracic kyphosis of < 10°), the Ponte osteotomy procedure was also effective in increasing the degree of thoracic kyphosis. Importantly, for patients in whom the thoracic kyphosis was within normal limits, the Ponte osteotomy procedure was effective in maintaining the physiological thoracic kyphosis after correction of the coronal alignment. Based on our experience, we propose that for the correction of spinal alignment in AIS, the multilevel Ponte osteotomy procedure provides a more effective and wider release of the structures of the posterior column than soft tissue release only, which facilitates the restoration and maintenance of the physiological kyphosis. The correction of thoracic kyphosis can be improved by the use of fully prebent titanium spinal rods, having a diameter > 5.5 cm, combined with the use of traditional distraction technology.

### Disadvantages of multilevel Ponte osteotomy procedure

In our case series, the Ponte osteotomy procedure increased operative time, as well as the volume of blood loss during surgery and the volume of blood needed for transfusion. Therefore, for patients with a poor physical condition or those at a greater risk of hemorrhaging during surgery, care should be taken to determine whether the Ponte osteotomy procedure would provide a distinct advantage over another procedure, including posterior release of the soft tissue only.

## Conclusion

In conclusion, for AIS deformities that have poor flexibility, the multilevel Ponte osteotomy procedure, combined with posterior selective segmental pedicle screw constructs, can provide a correction rate of the coronal and sagittal plane alignment that is comparable to that achieved for flexible AIS spines. The Ponte procedure improves the CCI and maintains the correction of thoracic kyphosis to a greater degree, ensuring the release of posterior tissues alone. However, the Ponte procedure increases operative time and volume of blood loss, issues that must be considered in the selection of the best procedure for a given patient. It is important to acknowledge the intermediate length of our follow-up period and the retrospective nature of our analysis, with large cohort, multicenter, randomized controlled trials to confirm the clinical effectiveness of the Ponte osteotomy procedure over other spinal correction procedures, including the release of the posterior soft tissues only.
